# Supplementary material for: SGTA associates with nascent membrane protein precursors
Source: EMBO Rep. 2020 Mar 25;21(5):e48835. doi: 10.15252/embr.201948835 (PMC7202230; doi:10.15252/embr.201948835)
Supplement: Supplementary file 1 — Appendix [file EMBR-21-e48835-s001.pdf]

APPENDIX

Appendix table of contents

Appendix Figure S1.
Photo-cross-linking of a ribosome-bound nascent chain page 2

Appendix Figure S2.
Identification of endogenous rabbit protein that interacts with nascent chains page 3

Appendix Figure S3.
SGTA and SGTB are highly conserved across mammalian species page 5

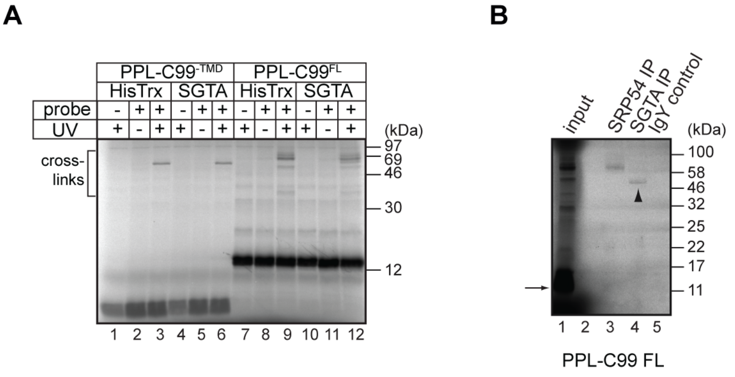

Appendix Figure S1. Photo-cross-linking of a ribosome-bound nascent chain

A PPL-C99$^{\text{-TMD}}$ and PPL-C99^{FL} lacking a stop codon were in vitro translated in the presence of 2 μM HisTrx or HisTrx-SGTA and either ε-TDBA-Lys-tRNA or tRNA-Lys. Reactions were irradiated with the UV light or left untreated, RNCs isolated, samples digested with RNaseA and resolved by SDS-PAGE followed by phosphorimaging analysis.

B PPL-C99^{FL} was in vitro translated in RRL in the presence of ε-TDBA-Lys-tRNA using an mRNA lacking a stop codon. Samples were irradiated with the UV light, RNCs isolated, samples digested with RNaseA and used in immunoprecipitation reactions with mouse anti-SRP54, chicken anti-SGTA or control chicken IgY antibodies. Samples were resolved by SDS-PAGE and results visualised by phosphorimaging. An arrowhead indicates a cross-linking adduct between PPL-C99^{FL} and endogenous rabbit SGTA whilst an arrow marks unmodified translation products.

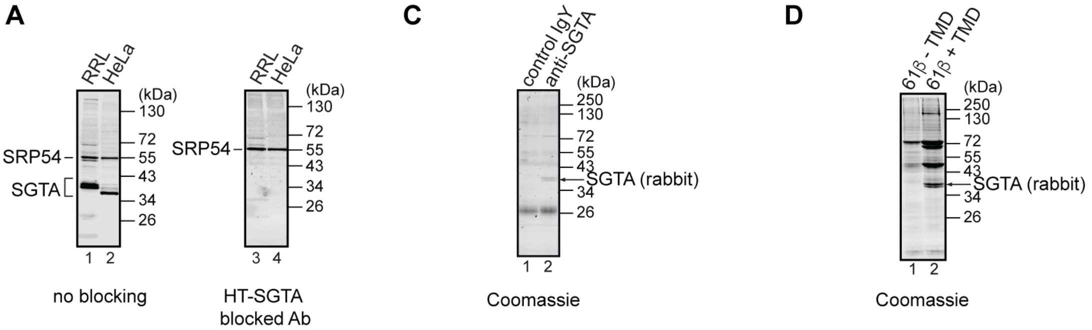

control IgY	Anti-SGTA Ab	
48.99	80.22	human SGTA
-	-	rabbit G1SX57

Score Sequest HT

Sec61β -TMD	Sec61β +TMD	
44.39	365.24	human SGTA
-	14.56	rabbit G1SX57

Score Sequest HT

B

```
G1SX57_RABIT MSSIKHLVYAVIRFLREQSQMDAYTSDEQESLEVAIQCLETVFKISSEDTHLAVPQPLTE 60
SGTA_HUMAN   MDNKKRLAYAIIQFLHDQLRHGGLSSDAQESLEVAIQCLETAFGVTVEDSDLALPQTLPE 60
             *.. *:*.**:*:**::* : .. :** *************.* :: **:.**:** * *

G1SX57_RABIT MFTNSFCKNDILPL------SNSAPEDVGKADQLKDEGNNHMKEENYAAAVDCYTQAIEL 114
SGTA_HUMAN   IFEAAATGKEMPQDLRSPARTPPSEEDSAEAERLKTEGNEQMKVENFEAAVHFYGKAIEL 120
             :*   :   :::             :  : ** .:*::** ***::** **: ***. * :****

G1SX57_RABIT DPNNAVYYCNRAAAQSKLSHYTDAIKDCEKAIAIDSKYSKAYGRMGLALTAMNKFEEAVT 174
SGTA_HUMAN   NPANAVYFCNRAAAYSKLGNYAGAVQDCERAICIDPAYSKAYGRMGLALSSLNKHVEAVA 180
             :* ****:****** ***.:*:.*::***:**.**  *************::**. ***:

G1SX57_RABIT SYQKALDLDPENDSYKSNLKIAEQKLREVSSPTGTGLSFDMASLINNPAFISMAASLMQN 234
SGTA_HUMAN   YYKKALELDPDNETYKSNLKIAELKLREAPSPTGGVGSFDIAGLLNNPGFMSMASNLMNN 240
              *:***:***:*::********* ****. ****   ***:*.*:***.*:***:.**:*

G1SX57_RABIT PQVQQLMSGMMTNAIG--GPAAGVGGLTDLSSLIQAGQQFAQQIQQQNPELIEQLRNHIR 292
SGTA_HUMAN   PQIQQLMSGMISGGNNPLGTPGTSPSQNDLASLIQAGQQFAQQMQQQNPELIEQLRSQIR 300
             **:*******::.. .  *   .   . .**:************:***********.:**

G1SX57_RABIT SRSFSSSTEEHS- 304
SGTA_HUMAN   SRTPSASNDDQQE 313
             **: *:*.:::.
```

Appendix Figure S2. Identification of endogenous rabbit protein that interacts with nascent chains

A HeLa cell lysate and RRL were resolved by SDS-PAGE and immunoblotted with the chicken anti-SGTA antibody that was either untreated ("no blocking") or pretreated with an excess of recombinant human HisTrx-SGTA ("HT-SGTA blocked Ab"). Anti-SRP54 immunoblotting was carried out in the same channel of infrared detection method and served as an exposure control between the two conditions.

B Sequence of human SGTA was aligned to the sequence of the closest annotated rabbit orthologue using Clustal Omega. "*" indicates fully conserved residues, ":" strongly similar amino acids and "." weakly similar amino acids. Sequences in red and blue indicate peptides corresponding to human SGTA and rabbit G1SX57, respectively, that were identified by mass spectrometry in experiments presented in panels C and D. Arrows indicate trypsin cleavage sites of the identified peptides.

C Chicken anti-SGTA antibody and control chicken IgY were covalently coupled to a solid support, incubated with RRL and bound proteins eluted at low pH. Eluates were resolved by SDS-PAGE, gel stained with Coomassie Brilliant Blue and used for in-gel tryptic digestion coupled with mass spectrometry based protein identification. Score Sequest HT for human SGTA and rabbit G1SX57 is shown.

D Bacterially expressed and purified Sec61β full-length and its variant lacking the TMD were immobilised on Ultralink Biosupport, incubated with RRL and proteins eluted in the presence of Triton X-100 were resolved by SDS-PAGE. Gel was stained with Coomassie Brilliant Blue and used for in-gel tryptic digestion and mass spectrometry based protein identification. Score Sequest HT for human SGTA and rabbit G1SX57 is shown.

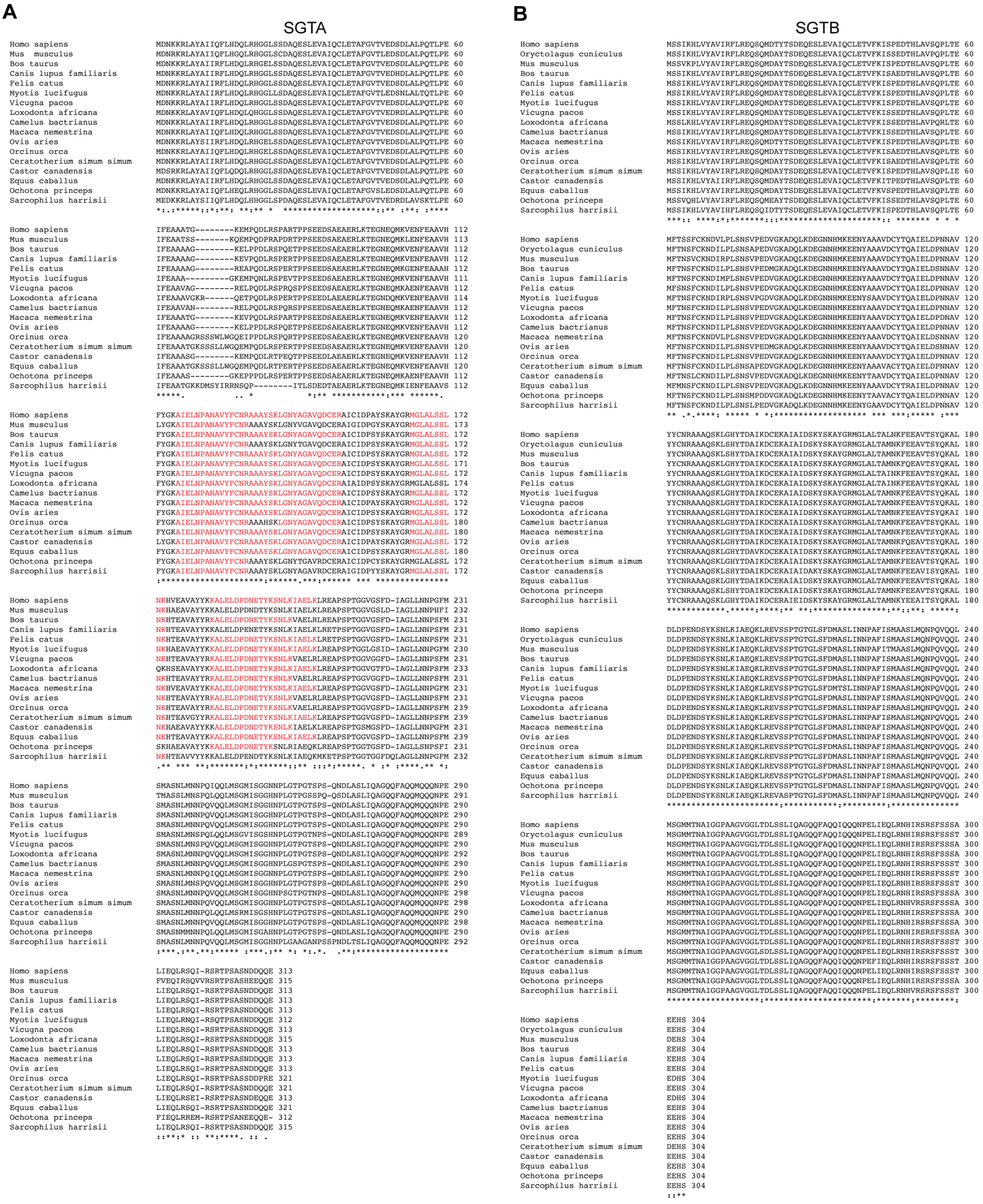

Appendix Figure S3. SGTA and SGTB are highly conserved across mammalian species

A, B Multiple sequence alignment of SGTA (A) and SGTB (B) from various mammalian species was carried out using Clustal Omega. Sequences in red indicate conserved peptides of human SGTA that were identified by mass spectrometry in experiments presented in Appendix Figs S2C and 2D. Homo sapiens – human, Oryctolagus cuniculus – rabbit, Mus musculus – house mouse, Bos taurus – cattle, Canis lupus familiaris – dog, Felis catus – domestic cat, Myotis lucifugus – little brown bat, Vicugna pacos – alpaca, Loxodonta africana – African savanna elephant, Camelus bactrianus – Bactrian

camel, Macaca nemestrina – pig-tailed macaque, Ovis aries – sheep, Orcinus orca – killer whale, Ceratotherium simum simum – southern white rhinoceros, Castor canadensis – American beaver, Equus caballus – horse, Ochotona princeps – American pika, Sarcophilus harrisii – Tasmanian devil.
