# Supplementary material for: SGTA associates with nascent membrane protein precursors
Source: EMBO Rep. 2020 Mar 25;21(5):e48835. doi: 10.15252/embr.201948835 (PMC7202230; doi:10.15252/embr.201948835)
Supplement: Supplementary file 2 — Expanded View Figures PDF [file EMBR-21-e48835-s002.pdf]

Expanded View Figures

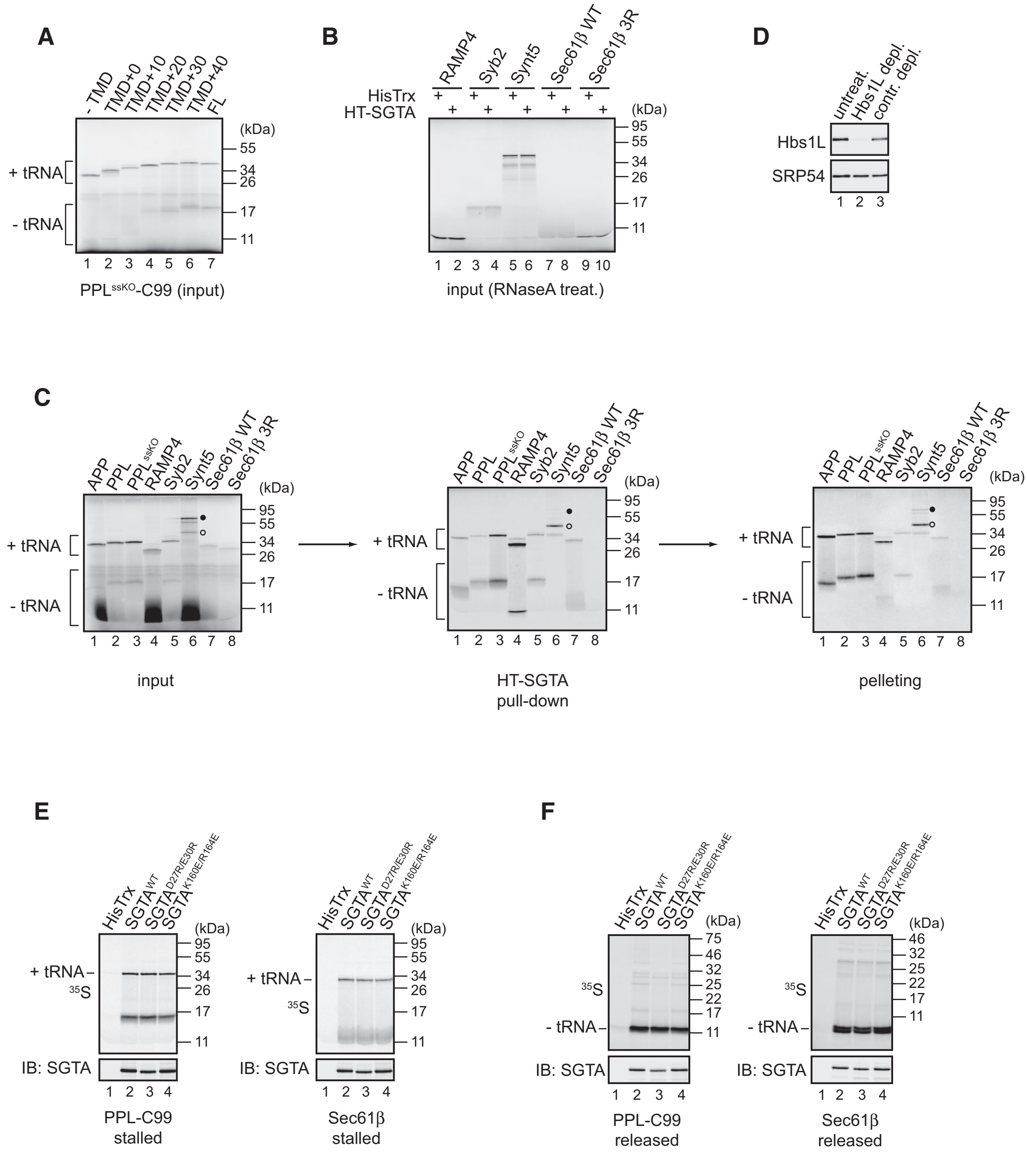

Figure EV1.

 © 2020 The Authors

Figure EV1. SGTA is recruited by hydrophobic ribosome-stalled nascent chains.

A Aliquots of total translation reactions of the indicated PPL^{ssKO}-C99 variants used for HisPur Cobalt resin pull-downs shown in Fig 3B were resolved by SDS–PAGE and results visualised by phosphorimaging. "−tRNA" and "+tRNA" indicate tRNA-lacking and tRNA-bound protein species, respectively.

B Aliquots of total translation reactions subsequently used for HisPur Cobalt resin pull-downs shown in Fig 3C were treated with RNaseA, samples resolved by SDS–PAGE and results visualised by phosphorimaging.

C Indicated full-length C99 variants (cf. Fig 1C) and TA proteins were translated in RRL from mRNAs lacking a stop codon in the presence of 2 μM HisTrx-SGTA ("input" panel). Reactions were incubated with HisPur Cobalt resin, bound proteins eluted at high imidazole concentration ("HT-SGTA pull-down" panel) and RNCs isolated by sedimentation through a sucrose cushion ("pelleting" panel). Samples were resolved by SDS–PAGE and results visualised by phosphorimaging. Samples shown in "HT-SGTA pull-down" panel correspond to ~ 20% of samples from "pelleting" panel. Open and filled circles indicate Synt5 species lacking or bound to tRNA, respectively.

D Rabbit reticulocyte lysates used in Fig 3D were immunoblotted with anti-Hbs1L antibody to establish the efficiency of Hbs1L immunodepletion. SRP54 was used as a loading control.

E, F PPL-$C99^{FL}$ and Sec61β were *in vitro* translated in RRL in the presence of 2 μM HisTrx or the indicated HisTrx-SGTA variants using mRNAs lacking (E) or containing (F) a stop codon. Reactions were incubated with HisPur Cobalt resin, bound proteins eluted at high imidazole concentration, samples resolved by SDS–PAGE and results visualised by phosphorimaging as described in Materials and Methods. Reactions were also immunoblotted with anti-SGTA antibody to ensure equal elution of the SGTA variants used.

Source data are available online for this figure.

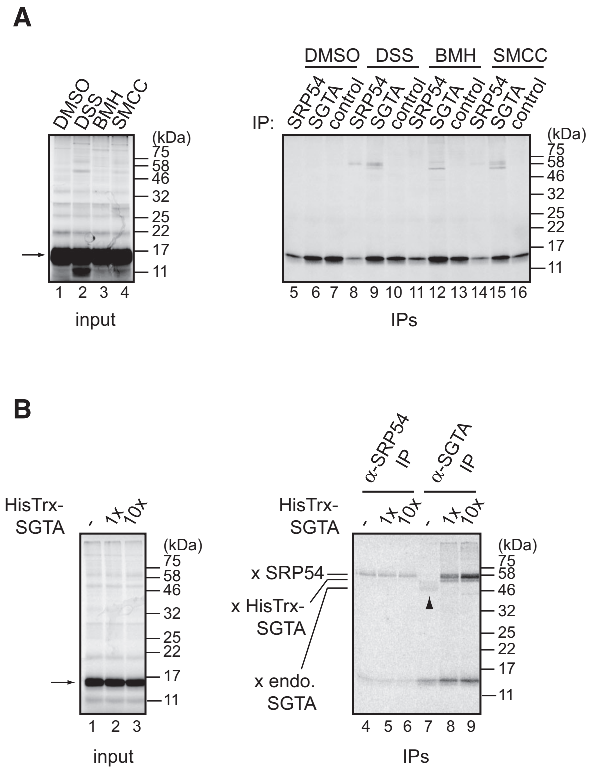

Figure EV2. Chemical cross-linking suggests a direct interaction between SGTA and a ribosome-stalled nascent chain.

A PPL-$C99^{FL}$ was *in vitro* translated in RRL in the presence of 2 μM HisTrx-SGTA using mRNA lacking a stop codon. Indicated cross-linkers were added, reactions incubated for 30 min at 10°C, and RNCs isolated and analysed by SDS–PAGE and phosphorimaging either directly (input) or following immunoprecipitation with anti-SRP54, chicken anti-SGTA or control chicken IgY antibodies. An arrow indicates unmodified translation products.

B As for panel (A) but DSS-mediated cross-linking was carried out in the presence of increasing concentrations of HisTrx-SGTA and adducts were immunoprecipitated with anti-SRP54 or chicken anti-SGTA antibodies. "1x" corresponds to 2 μM HisTrx-SGTA whilst "10x" to 20 μM HisTrx-SGTA. "x SRP54" and "x HisTrx-SGTA" indicate cross-linking adducts between stalled PPL-$C99^{FL}$ and endogenous SRP54 or recombinant HisTrx-SGTA, respectively. "x endo. SGTA" and an arrowhead indicate cross-linking of PPL-$C99^{FL}$ to endogenous rabbit SGTA. An arrow indicates unmodified translation products.

Source data are available online for this figure.

© 2020 The Authors

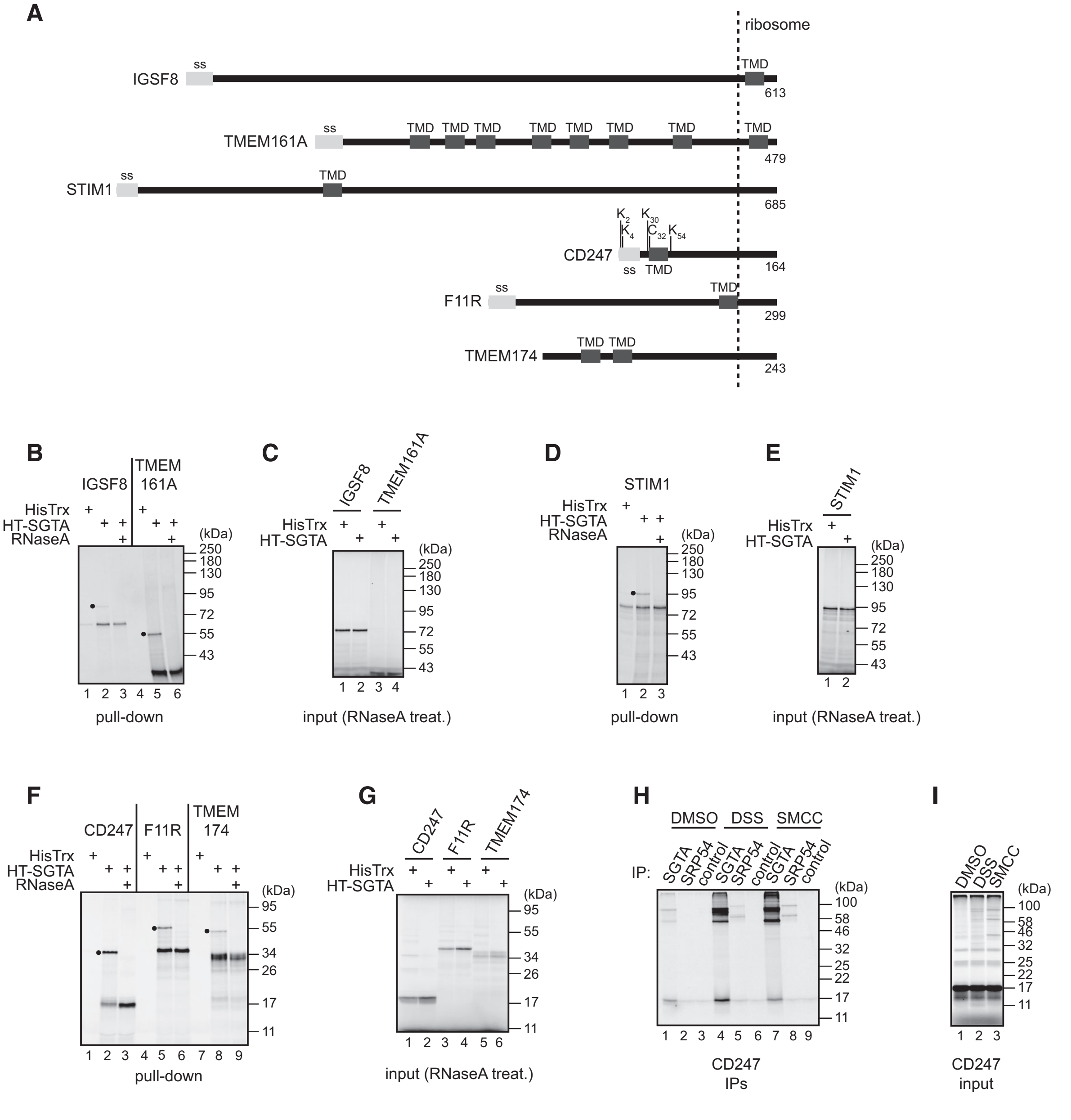

Figure EV3. SGTA binds co-translationally to a range of natural membrane proteins.

A A schematic representation of the proteins used in experiments presented in (B–I). Signal sequences and TMDs are shown in light and dark grey, respectively, and the amino acid length of nascent chains and 40 residues located inside the ribosomal exit tunnel are indicated. Lysine and cysteine residues located within or in the proximity of CD247 signal sequence and its TMD are shown. IGSF8—immunoglobulin superfamily member 8; TMEM161A—transmembrane protein 161A; STIM1—stromal interaction molecule 1; CD247—T-cell surface glycoprotein CD3 zeta chain; F11R—junctional adhesion molecule A; TMEM174—transmembrane protein 174.

B–G Indicated proteins were *in vitro* translated in RRL in the presence of 2 μM HisTrx or HisTrx-SGTA using mRNAs lacking a stop codon. Aliquots of the total translation reactions were digested with RNaseA and directly analysed by SDS–PAGE (input; C, E, G) whilst the remaining material was used in pull-down reactions carried out using HisPur Cobalt resin as described in Materials and Methods (B, D, F). High imidazole-eluted material from samples containing HisTrx-SGTA was split into two and either left untreated or digested with RNaseA, and all eluted fractions were analysed by SDS–PAGE followed by phosphorimaging. Filled circles indicate tRNA-bound protein species recovered with HisTrx-SGTA.

H, I CD247 was *in vitro* translated in RRL in the presence of 2 μM HisTrx-SGTA using an mRNA lacking a stop codon. Indicated cross-linkers were added, reactions incubated for 30 min at 10°C, and RNCs isolated and analysed by SDS–PAGE and phosphorimaging either directly (input, I) or following immunoprecipitation with anti-SRP54, chicken anti-SGTA or control antibodies (H). Faint CD247-SGTA adducts observed in the absence of any added cross-linking reagent likely result from the capacity of endogenous reticulocyte lysate factors to induce adduct formation in some cases as previously reported [69].

Source data are available online for this figure.

 © 2020 The Authors

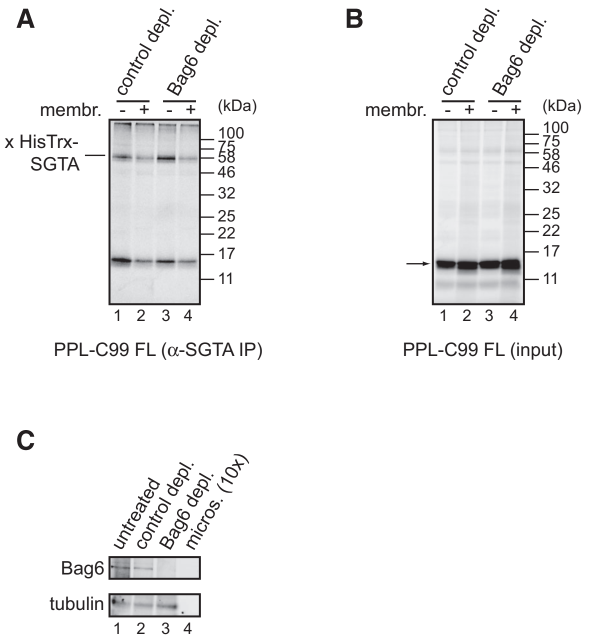

Figure EV4. Bag6 is dispensable for membrane-stimulated loss of SGTA.

A, B PPL-C99[FL] was *in vitro* translated in the presence of 2 μM HisTrx-SGTA using an mRNA lacking a stop codon and RRL depleted of Bag6 or control treated. DSS cross-linking reagent was added, reactions incubated for 30 min at 10°C, and RNCs isolated and analysed by SDS–PAGE and phosphorimaging either directly (input, B) or following immunoprecipitation with chicken anti-SGTA antibody (A). An arrow indicates unmodified translation products.

C The efficiency of Bag6 depletion from RRL and the presence of Bag6 at the ER-derived microsomes used in the experiment were tested by immunoblotting with anti-Bag6 and anti-tubulin antibodies. Note that 10× volume of microsomes relative to RRL used in the *in vitro* translation reactions was analysed.

Source data are available online for this figure.

© 2020 The Authors

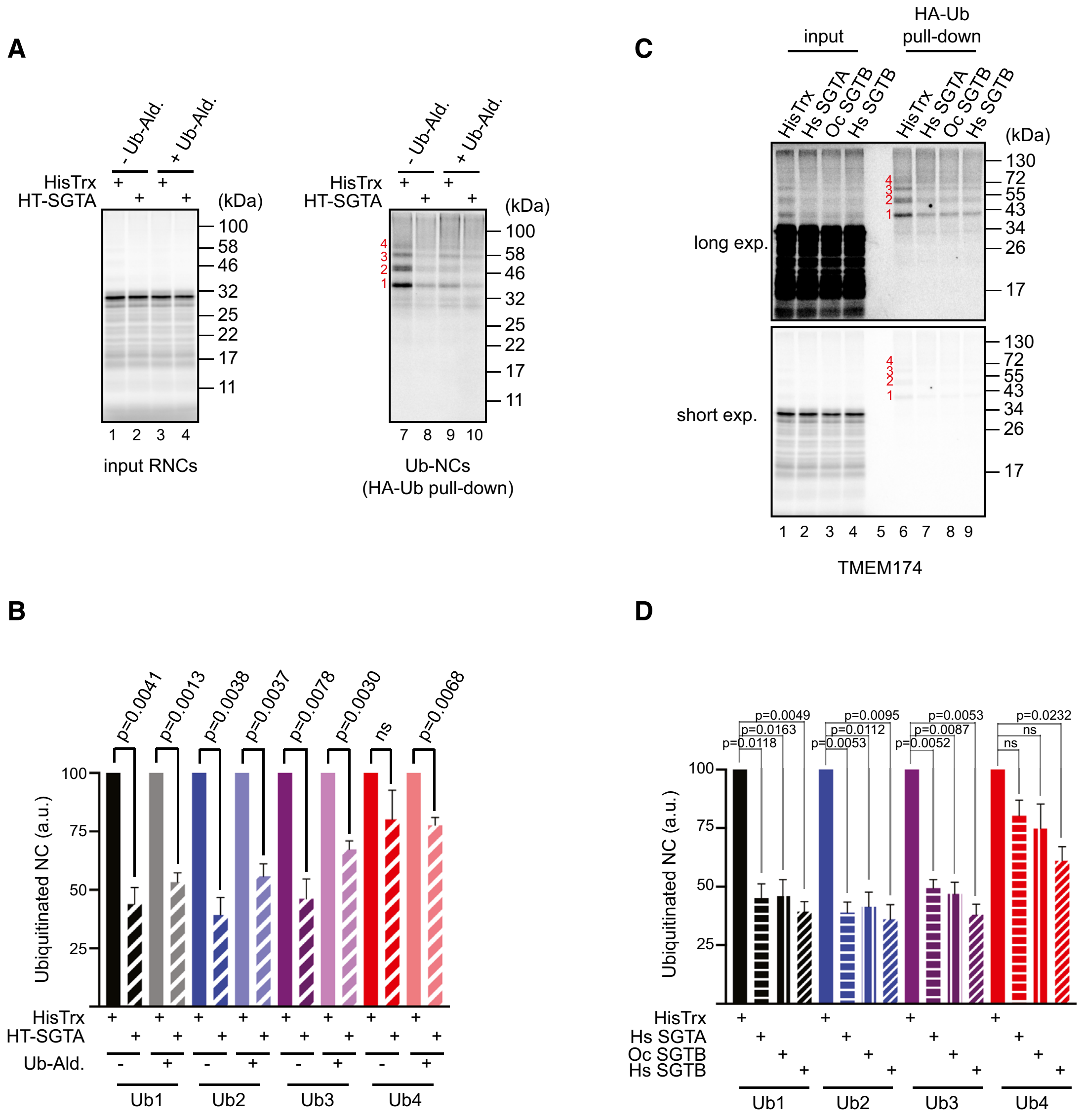

Figure EV5. SGTA-mediated nascent chain protection from ubiquitination does not rely on deubiquitinating enzymes and can be recapitulated by SGTB.

A, B TMEM174 was *in vitro* translated from an mRNA lacking a stop codon using RRL supplemented with 20 µM HA-tagged ubiquitin, 2 µM HisTrx or HisTrx-SGTA and 5 µM ubiquitin aldehyde (Ub-Ald) or control buffer. RNCs were isolated, ubiquitinated nascent chains recovered using anti-HA agarose, and samples resolved by SDS–PAGE and analysed by phosphorimaging (A). Individual ubiquitinated species of TMEM174 were quantified, and for each condition, their intensity was normalised to the value obtained for samples supplemented with HisTrx control protein (B). Shown is the mean with standard error of mean for $n = 4$ biological replicates. Statistical significance was calculated using unpaired t-test with Welch's correction. ns—not significant.

C, D Ubiquitination assay of stalled TMEM174 *in vitro* translated in the presence of 2 µM HisTrx, 2 µM human (Homo sapiens, Hs) HisTrx-SGTA, 2 µM rabbit (Oryctolagus cuniculus, Oc) HisTrx-SGTB or 2 µM human (Homo sapiens, Hs) HisTrx-SGTB was carried out as for panels (A and B), and Fig 7A (C). Individual ubiquitinated species were quantified and their intensity expressed relative to the samples supplemented with HisTrx control protein (D). Shown is the mean with standard error of mean for $n = 3$ biological replicates. Statistical significance was calculated using unpaired t-test with Welch's correction. ns—not significant.

Source data are available online for this figure.

 © 2020 The Authors
