# Supplementary figures and images for: SGTA associates with nascent membrane protein precursors
Source: EMBO Rep. 2020 Mar 25;21(5):e48835. doi: 10.15252/embr.201948835 (PMC7202230; doi:10.15252/embr.201948835)

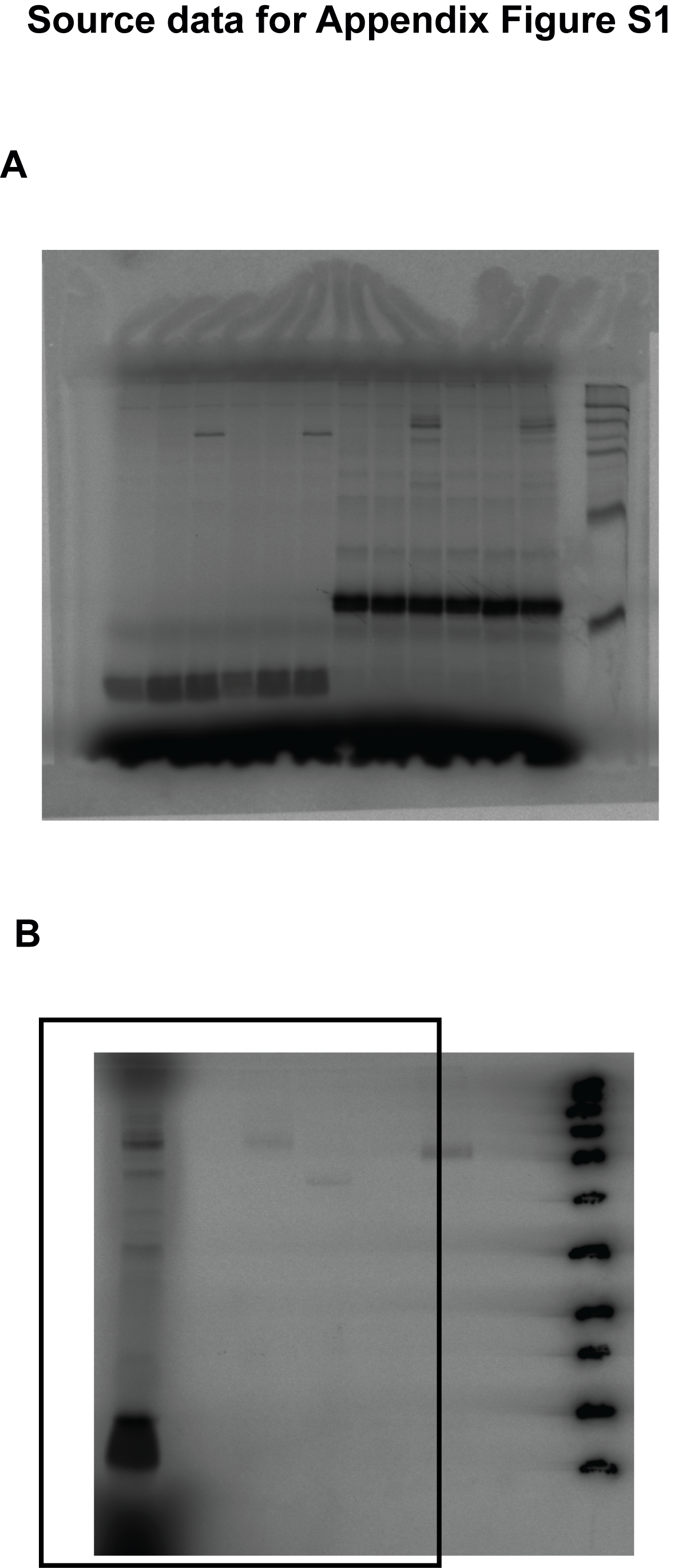

Supplement: Supplementary file 3 — Source Data for Expanded View and Appendix [file EMBR-21-e48835-s010.zip › EMBOR-2019-48835_SourceDataForAppendixFigureS1.tif]

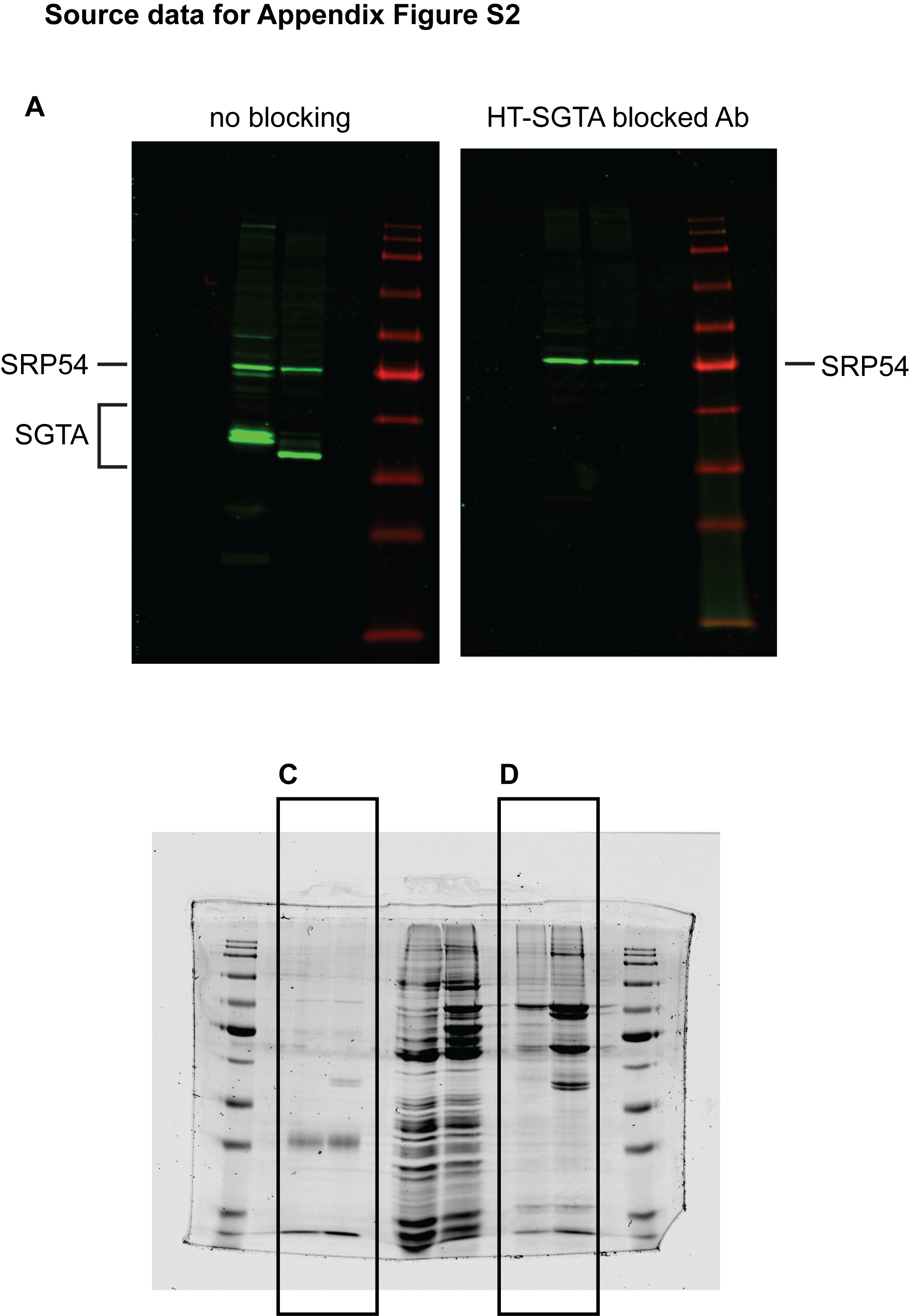

Supplement: Supplementary file 3 — Source Data for Expanded View and Appendix [file EMBR-21-e48835-s010.zip › EMBOR-2019-48835_SourceDataForAppendixFigureS2.tif]

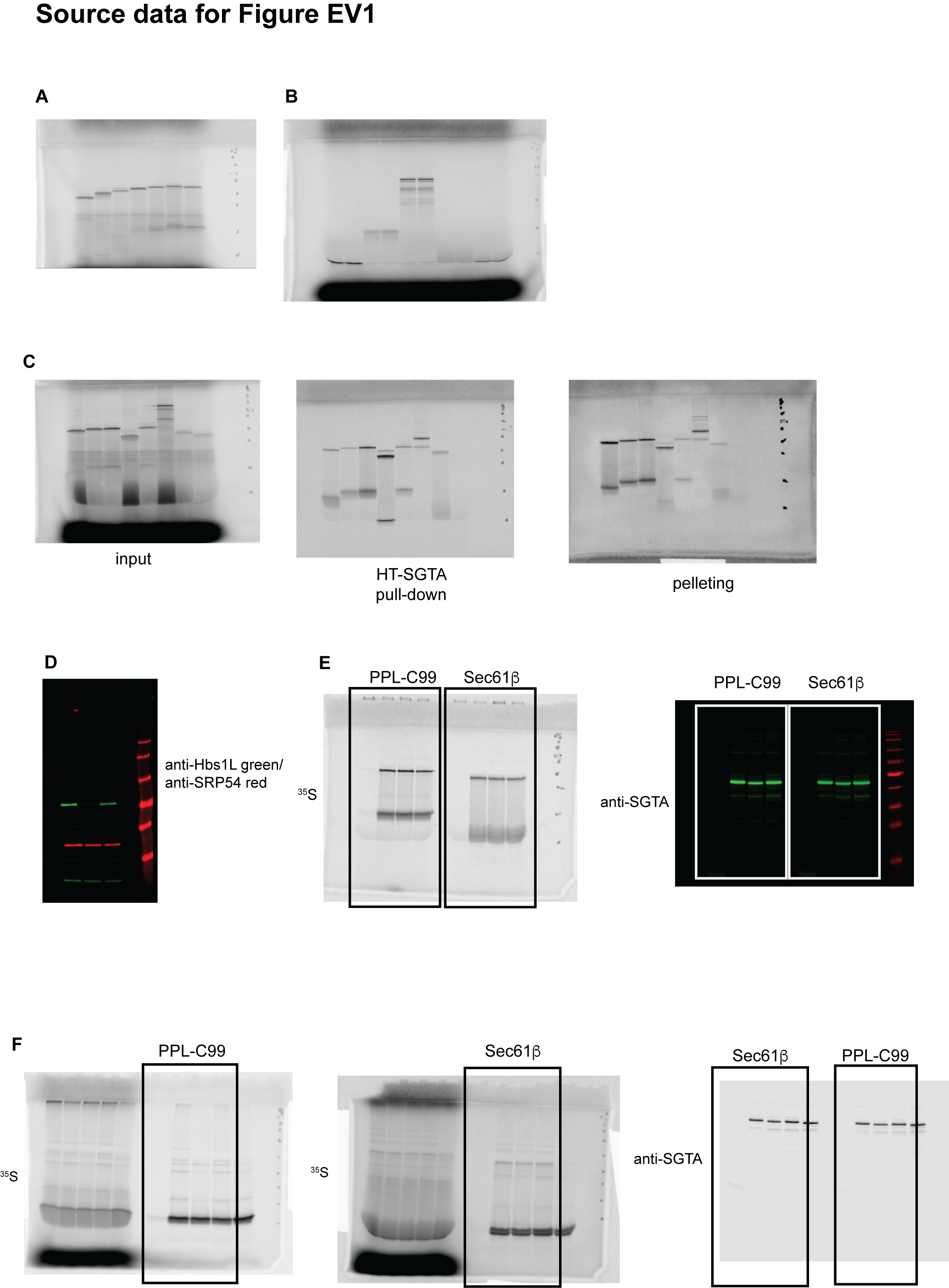

Supplement: Supplementary file 3 — Source Data for Expanded View and Appendix [file EMBR-21-e48835-s010.zip › EMBOR-2019-48835_SourceDataForFigureEV1.tif]

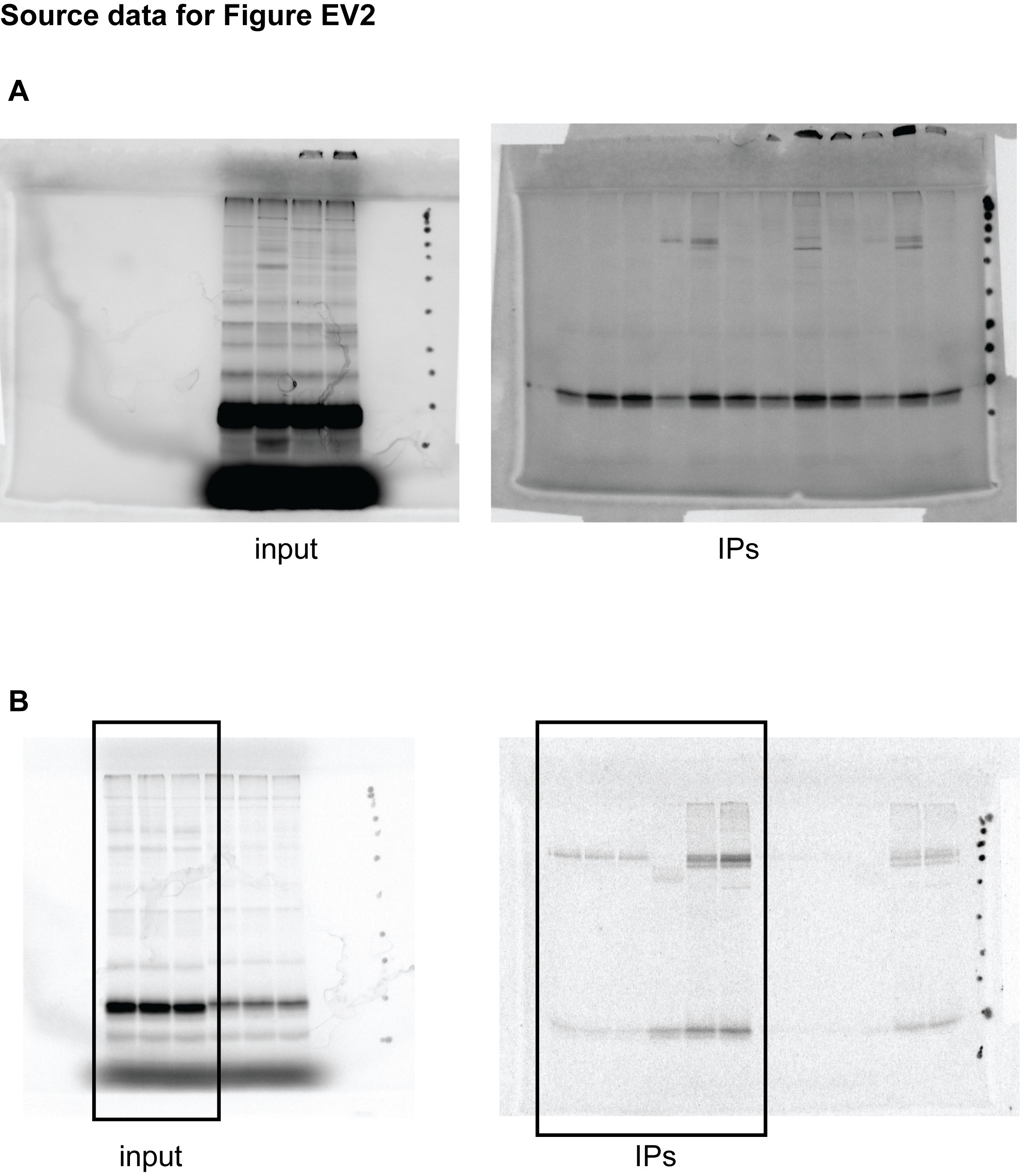

Supplement: Supplementary file 3 — Source Data for Expanded View and Appendix [file EMBR-21-e48835-s010.zip › EMBOR-2019-48835_SourceDataForFigureEV2.tif]

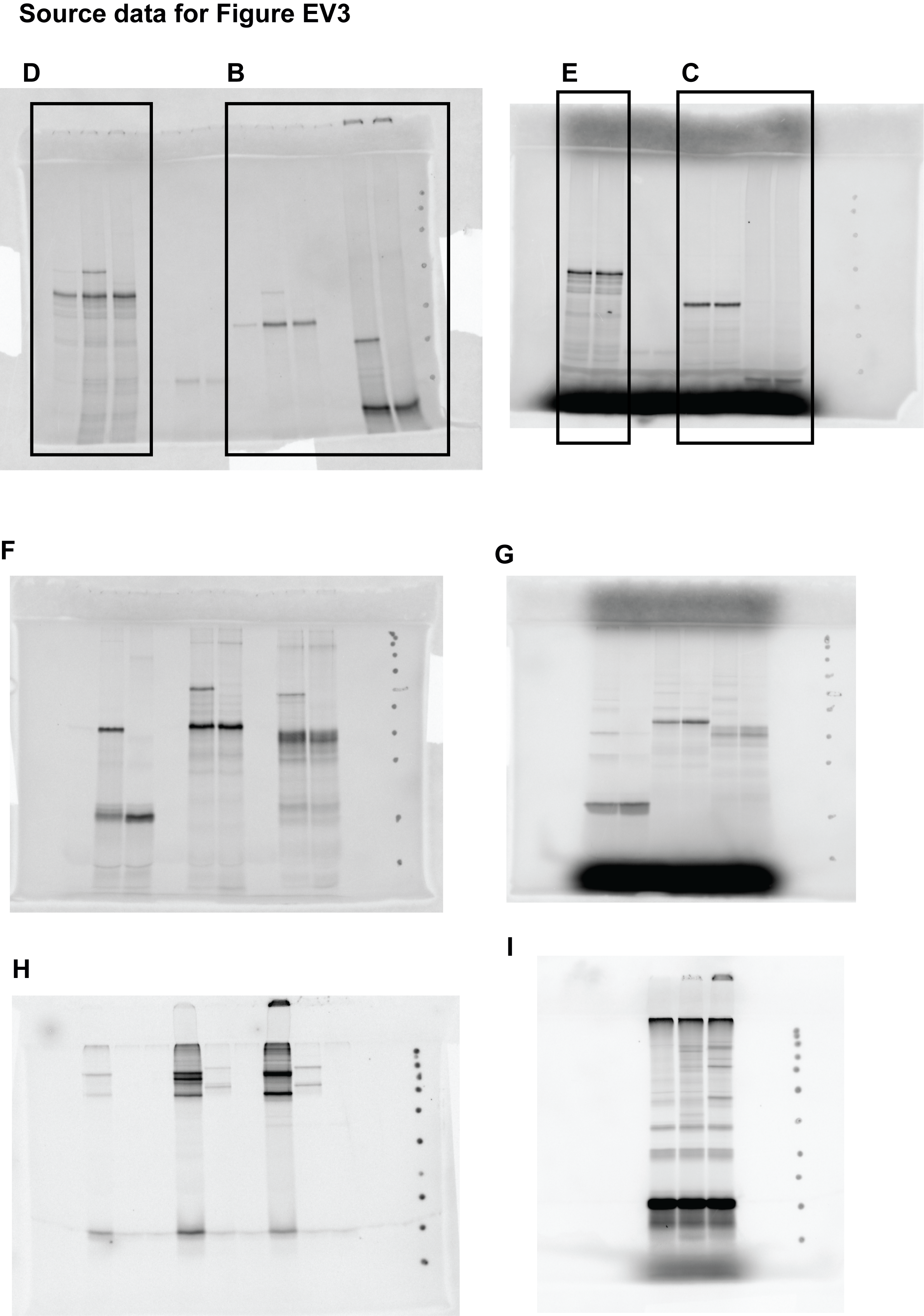

Supplement: Supplementary file 3 — Source Data for Expanded View and Appendix [file EMBR-21-e48835-s010.zip › EMBOR-2019-48835_SourceDataForFigureEV3.tif]

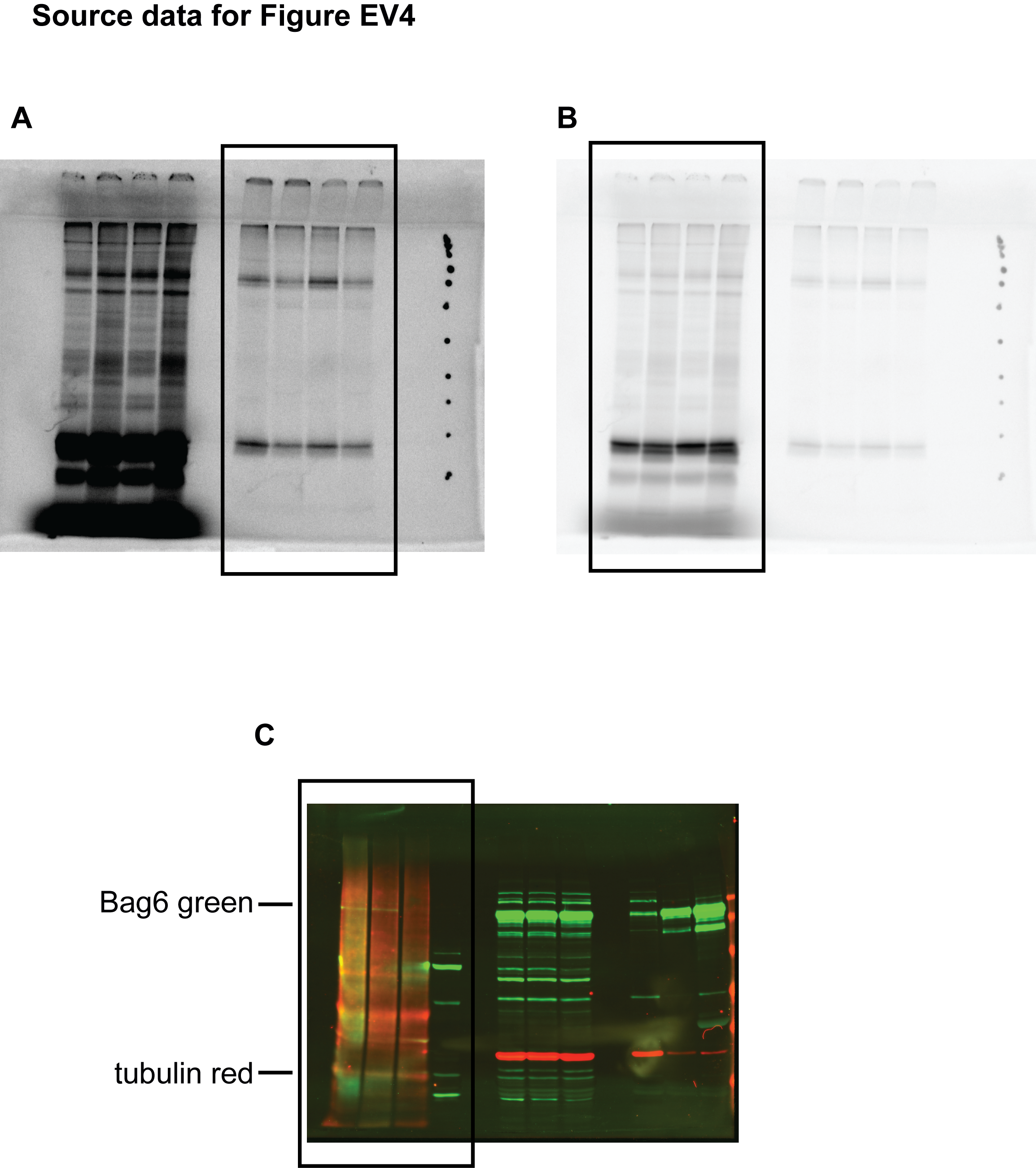

Supplement: Supplementary file 3 — Source Data for Expanded View and Appendix [file EMBR-21-e48835-s010.zip › EMBOR-2019-48835_SourceDataForFigureEV4.tif]

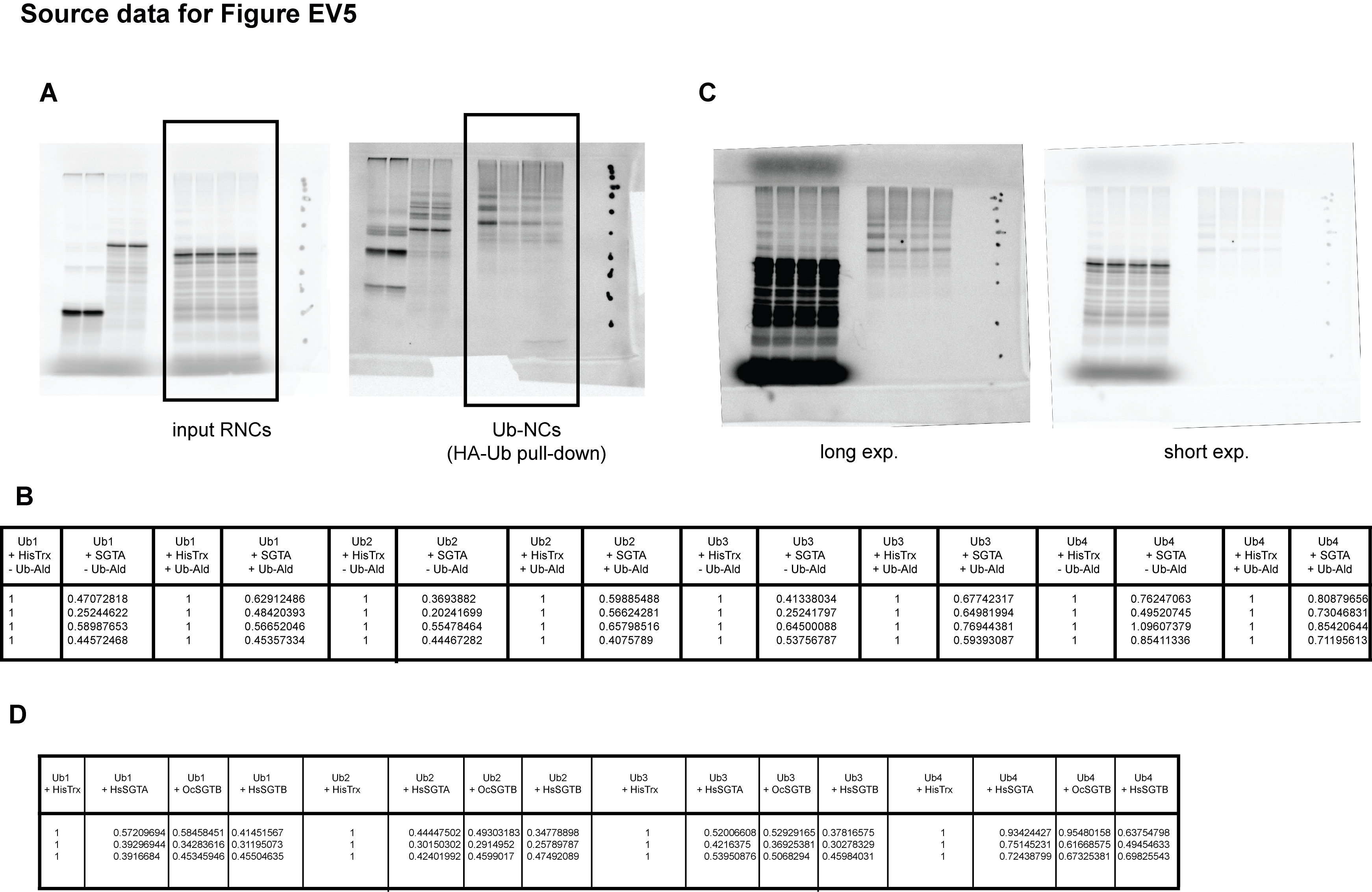

Supplement: Supplementary file 3 — Source Data for Expanded View and Appendix [file EMBR-21-e48835-s010.zip › EMBOR-2019-48835_SourceDataForFigureEV5.tif]
